# CD8^+^ T Cell Response to Gammaherpesvirus Infection Mediates Inflammation and Fibrosis in Interferon Gamma Receptor-Deficient Mice

**DOI:** 10.1371/journal.pone.0135719

**Published:** 2015-08-28

**Authors:** Brigid M. O’Flaherty, Caline G. Matar, Brian S. Wakeman, AnaPatricia Garcia, Carol A. Wilke, Cynthia L. Courtney, Bethany B. Moore, Samuel H. Speck

**Affiliations:** 1 Department of Microbiology and Immunology, Emory University School of Medicine, Atlanta, GA, United States of America; 2 Emory Vaccine Center, Emory University School of Medicine, Atlanta, GA, United States of America; 3 Division of Pathology, Yerkes National Primate Research Center, Emory University, Atlanta GA, United States of America; 4 Department of Internal Medicine, Division of Pulmonary and Critical Care Medicine, University of Michigan, Ann Arbor, MI, United States of America; Geisel School of Medicine at Dartmouth, UNITED STATES

## Abstract

Idiopathic pulmonary fibrosis (IPF), one of the most severe interstitial lung diseases, is a progressive fibrotic disorder of unknown etiology. However, there is growing appreciation for the role of viral infection in disease induction and/or progression. A small animal model of multi-organ fibrosis, which involves murine gammaherpesvirus (MHV68) infection of interferon gamma receptor deficient (IFNγR-/-) mice, has been utilized to model the association of gammaherpesvirus infections and lung fibrosis. Notably, several MHV68 mutants which fail to induce fibrosis have been identified. Our current study aimed to better define the role of the unique MHV68 gene, M1, in development of pulmonary fibrosis. We have previously shown that the M1 gene encodes a secreted protein which possesses superantigen-like function to drive the expansion and activation of Vβ4^+^ CD8^+^ T cells. Here we show that M1-dependent fibrosis is correlated with heightened levels of inflammation in the lung. We observe an M1-dependent cellular infiltrate of innate immune cells with most striking differences at 28 days-post infection. Furthermore, in the absence of M1 protein expression we observed reduced CD8^+^ T cells and MHV68 epitope specific CD8^+^ T cells to the lungs—despite equivalent levels of viral replication between M1 null and wild type MHV68. Notably, backcrossing the IFNγR-/- onto the Balb/c background, which has previously been shown to exhibit weak MHV68-driven Vβ4^+^ CD8^+^ T cell expansion, eliminated MHV68-induced fibrosis—further implicating the activated Vβ4^+^ CD8^+^ T cell population in the induction of fibrosis. We further addressed the role that CD8^+^ T cells play in the induction of fibrosis by depleting CD8^+^ T cells, which protected the mice from fibrotic disease. Taken together these findings are consistent with the hypothesized role of Vβ4^+^ CD8^+^ T cells as mediators of fibrotic disease in IFNγR-/- mice.

## Introduction

Fibroproliferative disorders are a class of diseases which result from dysregulated wound repair mechanisms, lead to excessive scaring and can affect multiple tissues and organ systems. Interstitial lung diseases (ILD), systemic and local scleroderma, liver cirrhosis, progressive kidney disease, cardiovascular disease, and macular degeneration are some of the fibrotic diseases affecting major organ systems [1]. Idiopathic pulmonary fibrosis (IPF), one of the most severe ILD, has unknown etiology and results in progressive scaring of lung tissue, respiratory failure, and eventual mortality. IPF affects middle-aged and elderly adults, occurring more frequently in males, and disease pathogenesis has been associated with a variety of environmental, genetic, and infectious factors (reviewed in [2–4]). Following clinical trials, two therapies (pirfenidone and nintedanib) were recently FDA approved [5, 6]; however, these therapies only delay functional decline. IPF has a median survival rate of 2–5 years post-diagnosis (reviewed in [7]). As such, a better understanding of the mechanisms driving disease is critical for developing better therapies.

To gain insights into the mechanisms driving fibrosis, researchers have focused on well-defined animal models of disease. Numerous small animal models exist for identifying mechanisms involved in driving pulmonary fibrosis (Reviewed in [8, 9]). MHV68 infection of IFNγR-/- mice has previously been shown to result in multi-organ fibrosis [10, 11], and has been highlighted as a potential model to study the role of gammaherpesvirus infections in development and exacerbation of IPF, due to pathologic and immunologic similarities to the disease in humans [12]. Key findings in this model have revealed roles for alternative macrophage activation, and the ability of MHV68 to induce epithelial to mesenchymal transition in the lung [13, 14]. Most strikingly, Mora and colleagues identified viral replication and reactivation as a critical driver of disease [15]. This study showed that inhibition of viral replication with a nucleoside analog, cidofovir, led to reduction in pathology and reversal of fibrosis. Further underscoring the importance of viral replication and persistence in disease, several latency compromised MHV68 mutant viruses failed to induce fibrotic disease in IFNγR-/- mice [16, 17].

We had previously identified the requirement for the unique, non-essential [18], MHV68 M1 gene product for the induction of multi-organ fibrosis in IFNγR-/- mice [19, 20]. We have previously shown that M1 functions as a novel viral superantigen, inducing the activation and expansion of Vβ4^+^ CD8^+^ T cells independent of antigen presentation [20]. During MHV68 infection, M1 plays an important role in suppressing viral reactivation from latently infected peritoneal macrophages, through activation and expansion of IFNγ producing Vβ4^+^ CD8^+^ T cells. As M1-null infected mice failed to develop fibrotic disease, we postulated that the Vβ4^+^ CD8^+^ T cell population induced during infection may contribute to lung pathology and fibrosis. Additional support for this hypothesis was lent by the observation that CD8^+^ T cells play a critical role in MHV68-induced fibrosis in the spleen [10].

Here we describe the immunologic changes observed during MHV68 infection of IFNγR-/- mice, comparing wild type (WT) MHV68 and an M1 null mutant (M1st). We show a strong correlation between inflammation and fibrosis, with heighted levels of inflammation and fibrosis observed in the M1 expressing virus infections. Additionally, we find striking M1-dependent changes to the cellular infiltrates in the lung, where elevated neutrophil and effector CD8^+^ T cell levels are observed in the presence of M1 expression. These data suggest that an M1-dependent alteration in cellular trafficking likely contributes to immunopathology. Notably we show that depletion of CD8^+^ T cells during the course of infection prevents fibrotic lung disease. Taken together, our data suggest a role for M1-induced Vβ4^+^ CD8^+^ T cells as mediators of fibrotic disease, where this population induces inflammation and altered cellular recruitment to the lung resulting in immunopathology and fibrosis.

## Results

### MHV68 mediated lethality in IFNγR-/- mice is associated with heightened inflammatory response to the virus

**Fig 1:**
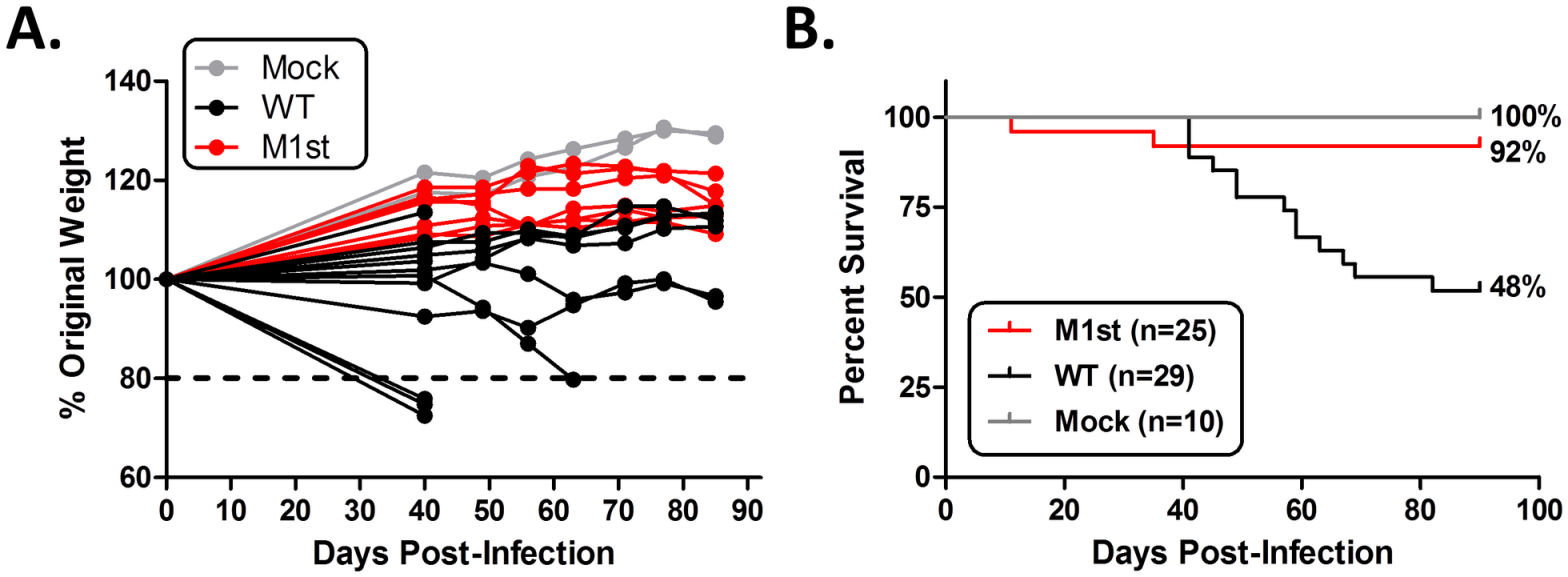
M1 expression is associated with lethality in MHV68 infected IFNγR-/- C57Bl/6 mice. 8–12 week old IFNγR-/- C57Bl/6 mice were intranasally infected with 1x10^5^ pfu MHV68 (WT or M1st) or were mock infected. (A) Mice were observed for weight change from starting weight, a loss of 20 percent or greater resulted in sacrifice. (B) Kaplan-Meier curves indicating mouse survival are shown. Significance was determined using log rank test with GraphPad software. *P* = 0.0027 for WT vs M1st infection.

**Fig 2:**
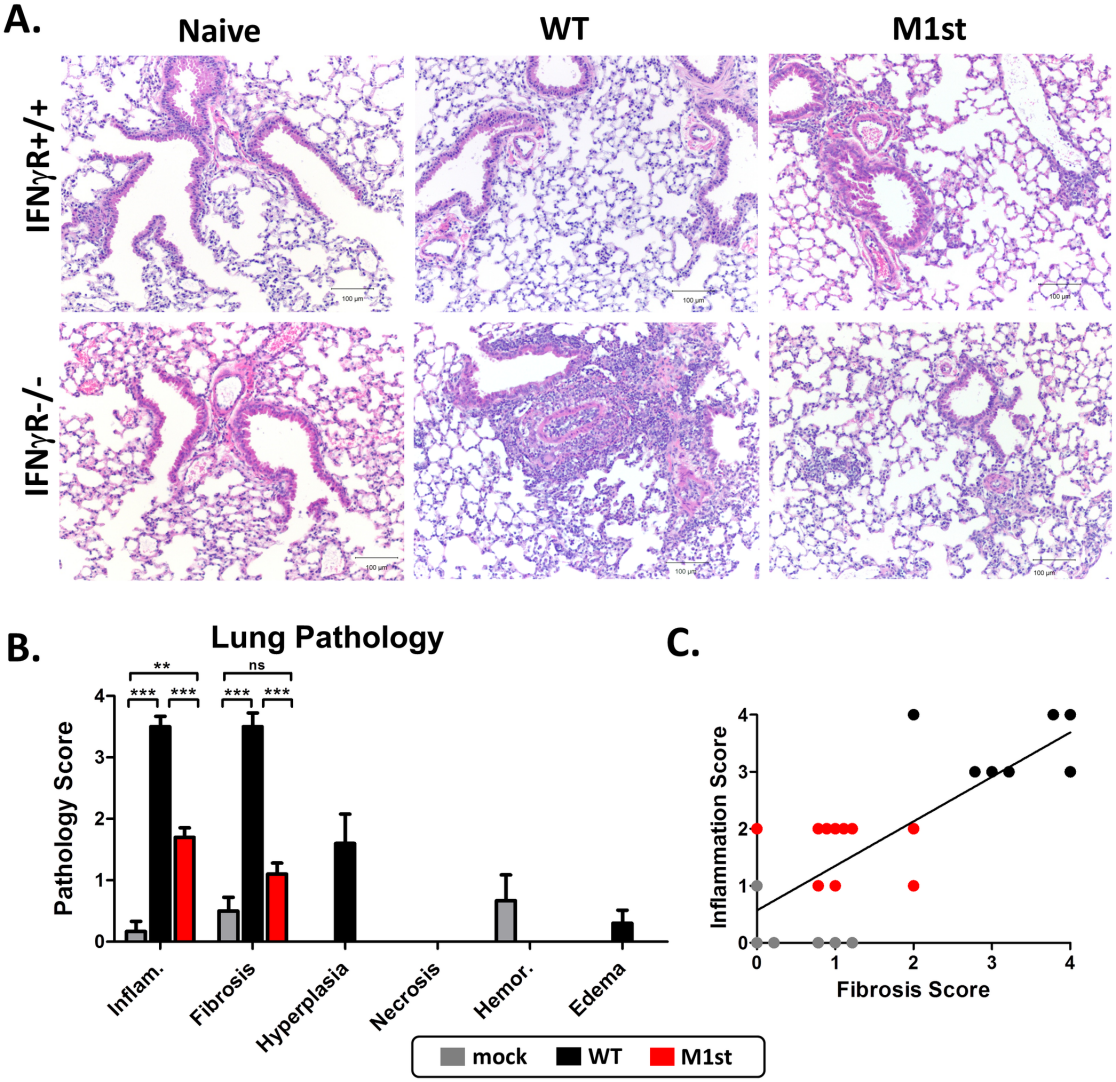
M1 induced fibrosis in IFNγR-/- C57Bl/6 mice is associated with heightened levels of inflammation in lung tissue. 8–12 week old WT or IFNγR-/- C57Bl/6 mice were intranasally infected with 1000 pfu MHV68 (WT or M1st) or were mock infected and sacrificed at 28 days post infection. Histological analysis was performed on lung tissues stained with hemotoxylin and eosin (H&E) or Masson’s Trichrome (MT). (A) Representative H&E stained sections are shown, scale bar = 100μm. Scores were determined for (A) pathology (mean and std. error are shown) from H&E sections and (B) fibrosis scores from MT sections, correlation was assessed using a Pearson’s Correlation test, showing R = 0.9929 at *P* = 0.0007, R^2^ = 0.9858. Mock (n = 6), WT (n = 10), and M1st (n = 10).

Lethality and multi-organ fibrosis are well characterized outcomes of MHV68 infection in IFNγR-/- C57Bl/6 mice. We have previously identified M1 as a critical viral factor involved in induction of fibrosis and lethality in this strain [19, 20]. Here we recapitulate this observation, showing that in the absence of M1 expression, mice are rescued from weight loss and subsequent lethality (Fig 1A and 1B). Protection from lethality appears to be associated with a reduction in pulmonary infiltrates (Fig 2A). M1-dependent pathology in IFNγR-/- mice includes dramatic changes to the lung architecture including thickening of the alveolar septa, type II pneumocyte hyperplasia, and extracellular matrix deposition (Fig 2A and 2B). Histological analyses revealed reduced levels of inflammation, fibrosis, hyperplasia, and edema in the M1st infected animals (Fig 2B). Notably, fibrotic foci were often associated with inflammatory infiltrates suggesting a causal role in lung damage and subsequent repair. This observation led to the identification of a striking correlation between inflammation and fibrosis in the lung (R = 1, *P* = 0.0167) (Fig 2C).

**Fig 3:**
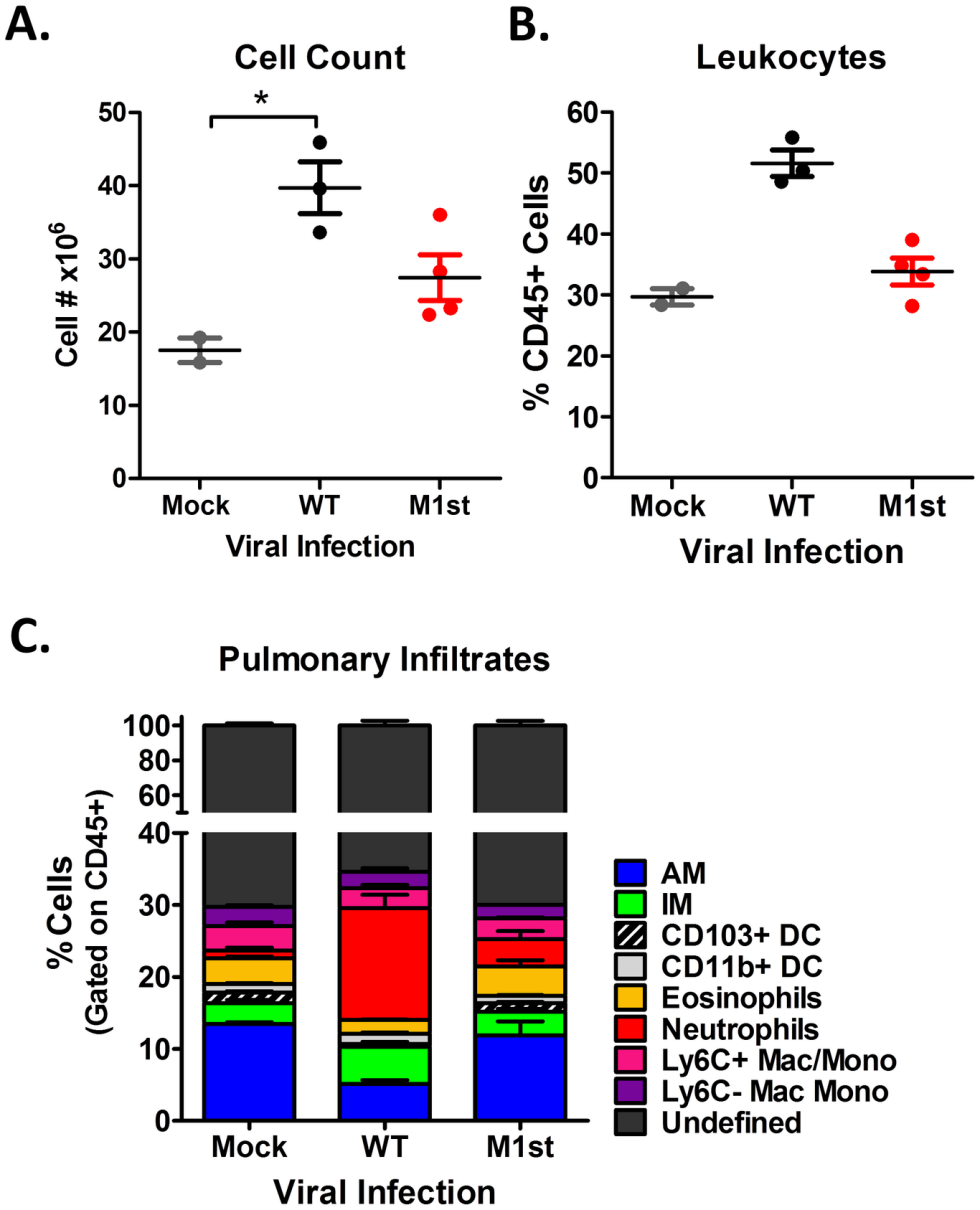
Global alterations in cellular composition of lung are observed in fibrotic IFNγR-/- C57Bl/6 mice. 8–12 week old IFNγR-/- C57Bl/6 mice were intranasally infected with 1x10^5^ pfu MHV68 (WT or M1st) or were mock infected and sacrificed at 28 days post infection. Whole lungs were assessed for (A) total number of cells (mean and std. error shown), (B) frequency of innate leukocytes (mean and std. error), and (C) frequency of innate populations (described in [21]). Statistics were performed using Kruskal-Wallis test with Dunn’s post test, *P* = 0.0463. Mock (n = 2), WT (n = 3), M1st (n = 4).

To characterize the inflammatory response in the lungs of infected IFNγR-/- mice, we utilized a panel of makers described by Misharin *et al*. [21], to identify innate cell populations by flow cytometry. As a result of infection, both M1st and WT viruses resulted in increased lung cellularity at 28 days post-infection, suggesting increased recruitment of leukocytes to the lung (Fig 3A). However, we note that WT infected mice showed a more striking increase in cellularity when compared to the moderate increase observed in M1st infected animals. To identify cellular subsets in the lung, we gated on leukocytes (CD45^+^ cells) and observed only slight differences in the frequencies of leukocytes among the different infection conditions (Fig 3B). Pulmonary infiltrates were classified as macrophages, dendritic cells, eosinophils, neutrophils, and monocytes based on surface marker expression. Here we observed a striking M1-dependent increase in the frequency of neutrophilic infiltrates and a reduction in alveolar macrophages (Fig 3C). Over the course of infection differences in the M1-dependent infiltrating populations were shown to be most striking between 18 and 28 days post-infection (S1 Fig). Consistent with previous observations [13], we observed an increase in absolute numbers of alveolar macrophages during MHV68 infection (S2A Fig). As alternative macrophage activation (M2) has been described as a potential driver of fibrotic disease, we were surprised to note that M1st and WT infected IFNγR-/- mice had similar numbers of alveolar macrophages with an M2 phenotype (Fizz1^+^ and CD206^+^). We did observe a slight elevation in the number of interstitial macrophages with an M2 phenotype by 28 days post-infection in WT, but not M1st infected mice—however, this failed to reach statistical significance (S2B Fig).

### Failure to control viral persistence is not sufficient to induce inflammation and fibrosis

**Fig 4:**
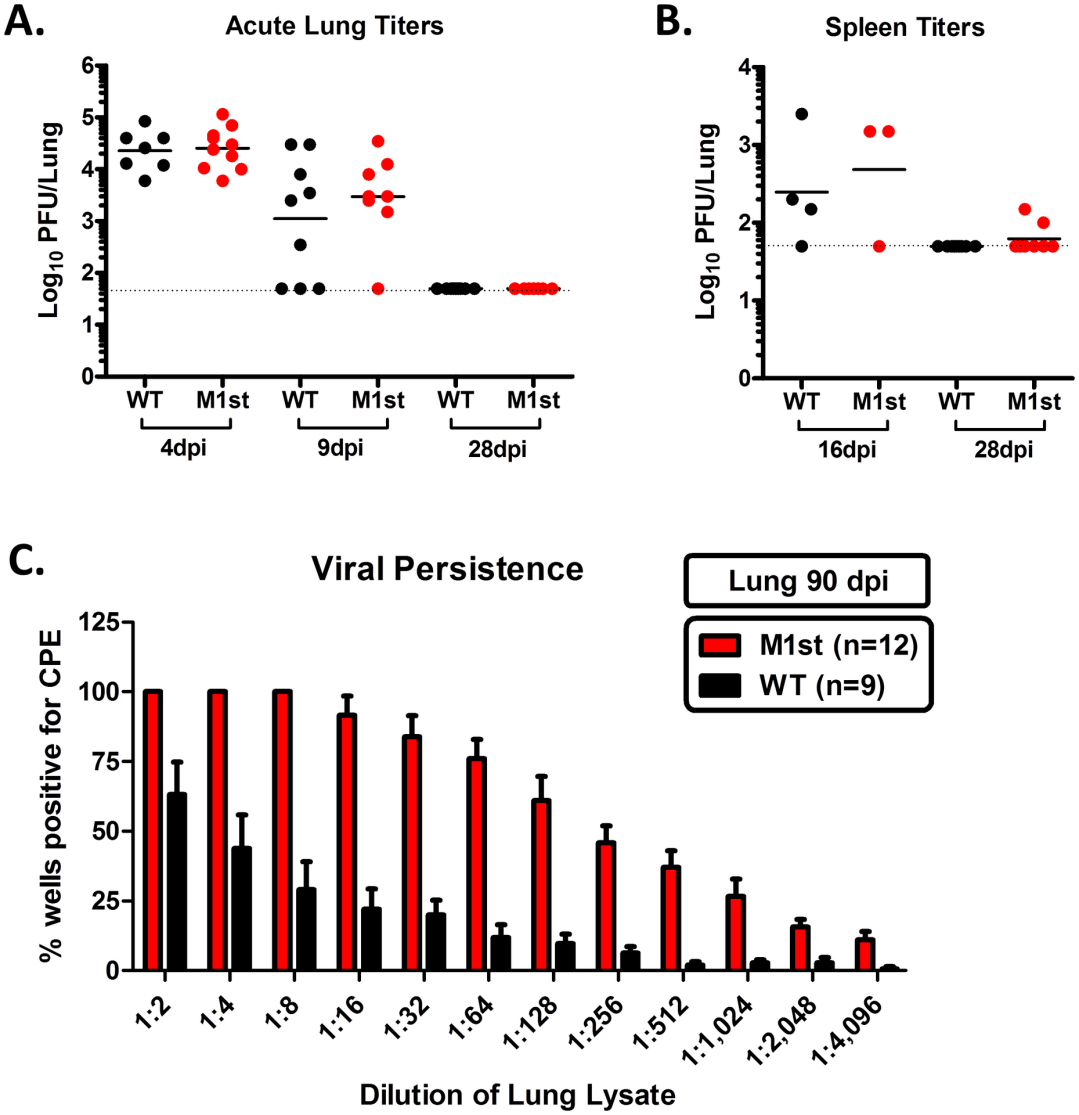
The absence of M1 expression does not impact acute viral replication, and M1 is not required for viral persistence in the lung. 8–12 week old IFNγR-/- C57Bl/6 mice were intranasally infected with 1x10^5^ pfu MHV68 (WT or M1st) and sacrificed at indicated times. (A) Lung titers from mice at days 4, 9, and 28 post infection (n = 7–10 mice/group from two independent experiments). (B) Spleen titers from mice at days 16 and 28 days post infection (n = 4–9 mice/group one or two independent experiments). (C) Viral persistence was measured by assessing cytopathic effect (CPE) induced by infected lung lysate plated on mouse embryonic fibroblasts (mean and std. error are shown).

Although M1 expression is dispensable for the establishment of latency in wild type C57Bl/6 mice [18], our data raised the possibility that the reduction in M1st virus immunogenicity in IFNγR-/- mice could simply be due to a defect in virus replication. To address this possibility we evaluated acute titers in the lung and spleen at various times post-infection. As anticipated, we find that M1 is dispensable for acute replication in the lungs and spleen of IFNγR-/- mice (Fig 4A and 4B). However, since it has previously been noted that viral reactivation and persistent replication are critical for development of MHV68-induced fibrotic disease [16], we next assessed persistent virus replication in the lungs at a late time point in WT and M1st infected IFNγR-/- mice (Fig 4C). Notably, we observed higher levels of persistent virus replication at 90 days post-infection in the lungs of IFNγR-/- mice infected with M1st than in those infected with WT MHV68. This strongly argues that persistent virus replication alone is not sufficient to induce a fibrotic response (Fig 4C). It should be noted that the levels of viral persistence were measured from surviving mice; and as such these values may under-represent the amount of persistent virus replication in WT infected mice (in which have observed ca. 50% lethality).

### Absence of M1 expression does not alter expression of the profibrotic mediator TGFβ in lung

**Fig 5:**
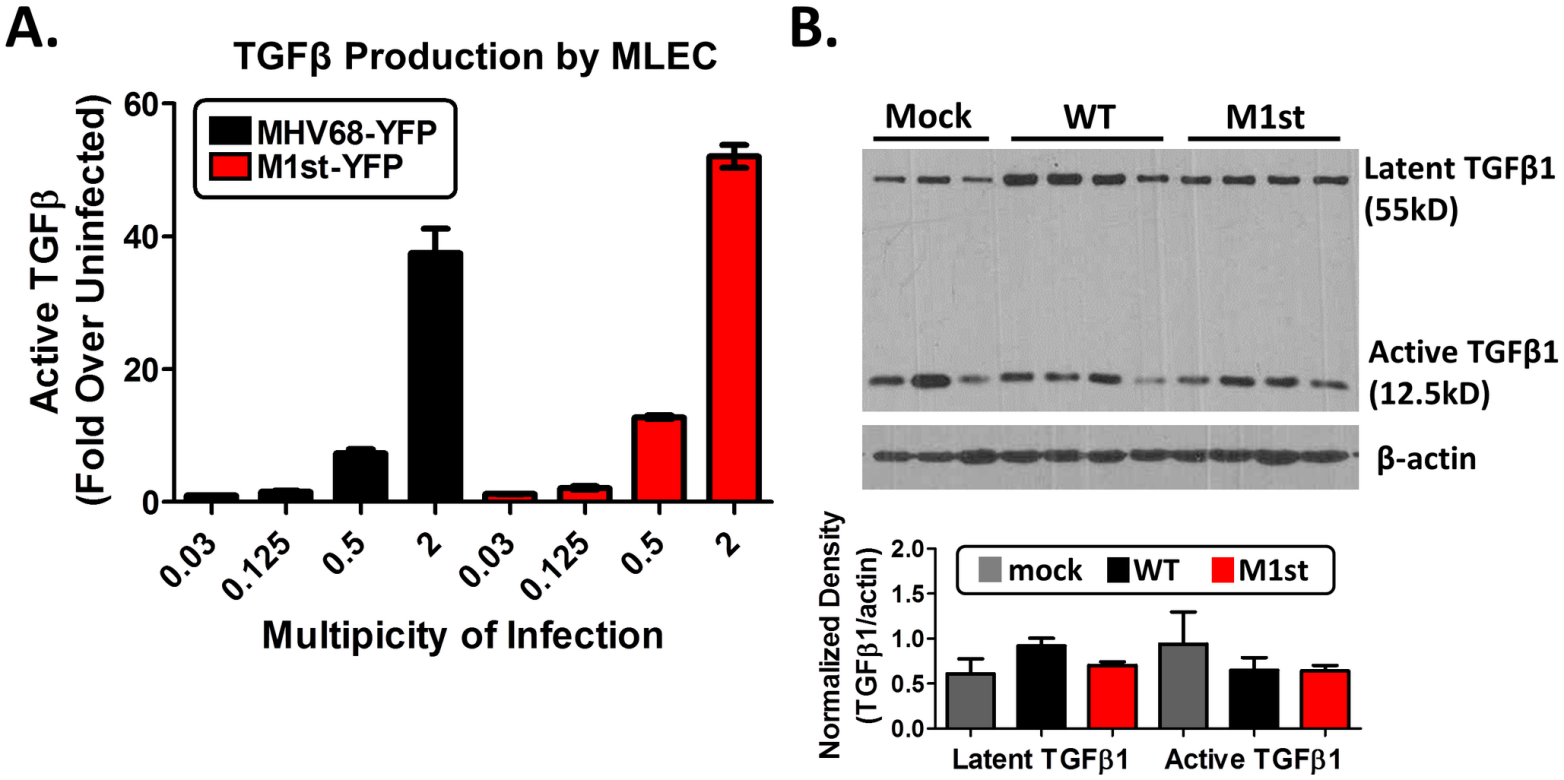
The absence of fibrosis in M1st infected IFNγR-/- C57Bl/6 mice is not due to a failure in profibrotic mediator TGFβ1 expression. (A) Mink lung epithelial cells (MLEC-clone 32) stably transfected with a plasminogen activator inhibitor-1 fused to a luciferase reporter gene were infected with differing multiplicities of infection and assayed for luciferase activity (a read out of active TGFβ production). 8–12 week old IFNγR-/- C57Bl/6 mice were intranasally infected with 1x10^5^pfu MHV68 (WT or M1st) or mock infected and sacrificed at 28 days post-infection (n = 3–4 mice/group). (B) lung lysates (30μg) were assessed for TGFβ1 expression by western blot (mock lane 1–3, WT lane 4–7, M1st lane 8–11), (C) normalized band density is shown.

Numerous fibrotic diseases are mediated by the cellular growth factor and cytokine transforming growth factor beta (TGFβ) (reviewed [22]). MHV68 infection has been shown to induce TGFβ production from a variety of cell types *in vivo* [23], and like EBV [24], MHV68 infection of alveolar epithelial cells *in vitro* and *in vivo* results in TGFβ production [25]. To determine whether the lack of a fibrotic response in M1st infected mice was due to a defect in TGFβ induction, we evaluated the ability of WT and M1st MHV68-YFP viruses to induce active TGFβ in mink lung epithelial cells stably transfected with a plasminogen activator inhibitor-1 (PAI1) luciferase reporter [26]. In this cell line the TGFβ responsive PAI1 promoter is fused to a firefly luciferase gene, and presence of active TGFβ results in luciferase expression. Testing various multiplicities of infection, we find that the M1st virus induced levels of TGFβ similar to the WT virus (Fig 5A). To verify that the M1st virus was capable of inducing equivalent levels of TGFβ *in vivo*, we evaluated lung homogenates from mice at 28 days post-infection. We find roughly equivalent levels of latent and active TGFβ in M1st and WT infection—although notably these were not significantly different from the levels observed in lungs homogenates recovered from mock infected animals (Fig 5B).

### Absence of fibrosis is associated with reduced CD8^+^ T cell responses during infection

**Fig 6:**
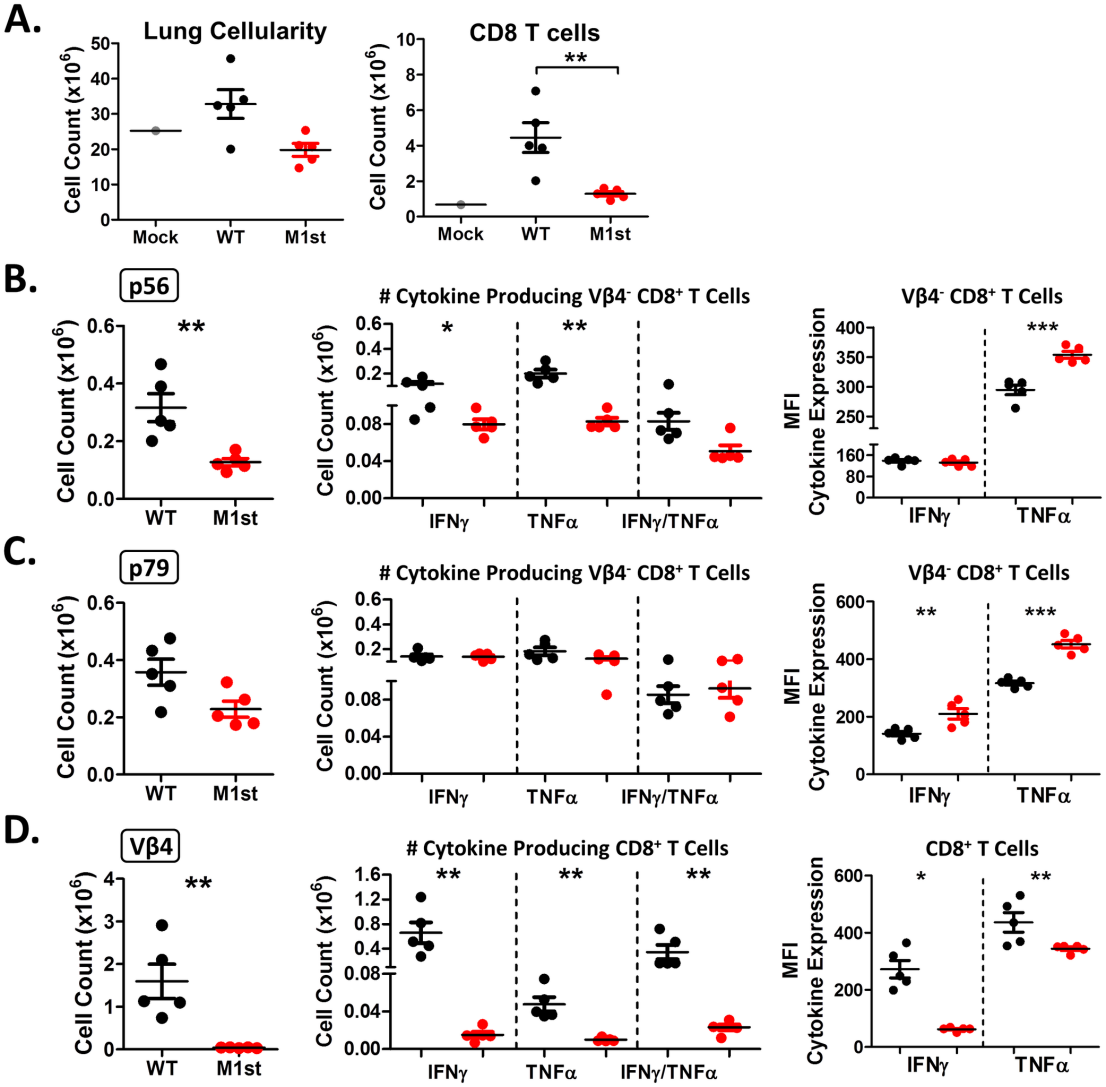
Reduced CD8^+^ and effector CD8^+^ T cells are observed in absence of M1 expression. 8–12 week old IFNγR-/- C57Bl/6 mice were intranasally infected with 1x10^5^ pfu MHV68 (WT or M1st) and sacrificed at 28 days post infection. Whole lungs were harvested and assessed for presence of CD8^+^ T cell populations and effector function (n = 5 mice/group). Mean and std. error are shown for (A) absolute number of lung and CD8^+^ T cells, (B&C) absolute number of tetramer specific and peptide responsive CD8^+^ T cells, with MFI of cytokine expression, (D) absolute number of Vβ4^+^ CD8^+^ T cells and M1 responsive CD8^+^ T cells. Statistics were measured using a Mann-Whitney 2 tailed test (* *P* = 0.0119, ** *P* = 0.0079, * *P* = <0.0004).

Viral specific T cell response during MHV68 infection is characterized by a heterogeneous population of epitope specific CD8^+^ T cells which have two dominant patterns of expansion and contraction [27–29]. During MHV68 infection the H-2Db epitope restricted “pattern 2” responders, exemplified by p56 (ORF6), arise and decline rapidly in response to acute replication [27, 30]. The H-2Kb epitope restricted “pattern 1” responders, exemplified by p79 (ORF61), arise rapidly but have a more gradual decline maintaining relatively high frequencies late into infection [27, 30]. Pattern 1 responders are thought to develop in response to viral antigen expressed during reactivation from latently infected B cells [31, 32].To evaluate both viral antigen-specific T cell response and Vβ4^+^ CD8^+^ T cell expansion, we assessed these populations at 28 days post-infection. As a measure of efficiency of CD8^+^ T cell response during infection, we measured levels of epitope specific CD8^+^ T cells and response to their cognate peptides. Our analyses show a significant reduction in overall CD8^+^ T cells levels in the absence of M1 expression (Fig 6A). More strikingly, we noted reduced epitope specific CD8^+^ T cell numbers in M1st infected mice, using tetramer staining for p56 and p79 specific T cells (Fig 6B and 6C), suggesting that recruitment of these cells to the lung is less efficient. We find decreased levels of responsiveness in p56 but not p79 specific CD8^+^ T cells in the M1st infection compared with WT infection (Fig 6B and 6C), perhaps reflecting the different kinetics of these effector populations. However, a notable increase in cytokine production was observed in the absence of M1 expression, with p56 responsive cells producing more TNFα and p79 responsive cells producing more IFNγ and TNFα –suggesting that there is no inherent defect in ability to produce cytokines, and that they may produce these cytokines even more efficiently (Fig 6B and 6C). The latter observation could be driven by higher levels of persistent virus replication in the lungs of M1st infected IFNγR-/- mice, as shown in Fig 4C.

Concomitant with the establishment of MHV68 latency is the M1-dependent Vβ4^+^ CD8^+^ T cell expansion which occurs between 21 and 28 days post-infection [33]. This population of cells is thought to control MHV68 viral reactivation in C57Bl/6 mice, through the secretion of IFNγ [19, 20]. Following expansion, Vβ4^+^ CD8^+^ T cells remain elevated though the life of infection, requiring continued stimulation by the stimulatory ligand, M1, in a MHC independent manner [20, 33, 34]. As anticipated the expanded Vβ4^+^ CD8^+^ T cell population in WT, but not M1st, infected mice were capable of producing IFNγ and TNFα in response to recombinant M1 protein (Fig 6D).

**Fig 7:**
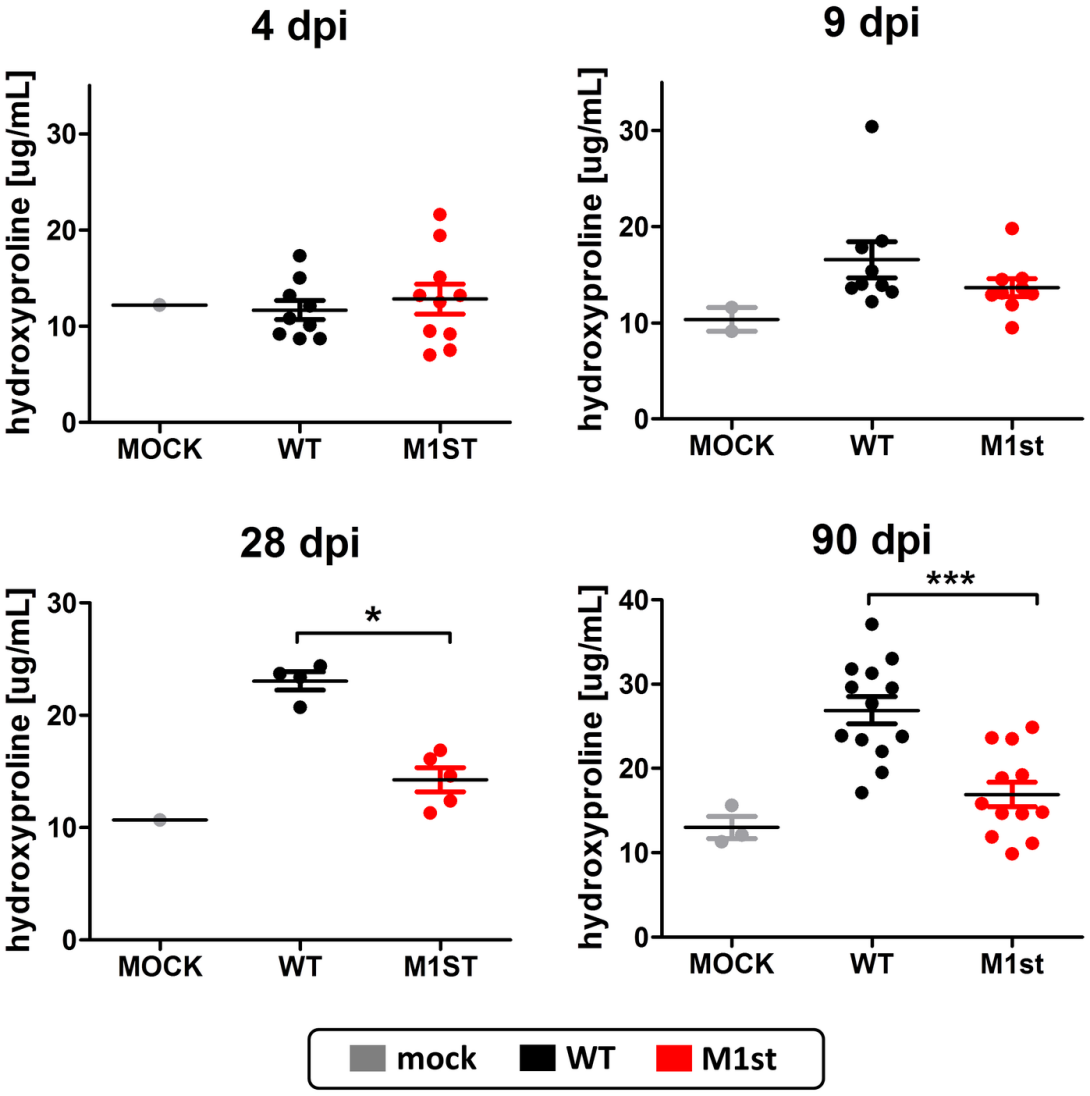
Development of M1 dependent fibrosis in IFNγR-/- C57Bl/6 mice correlates with timing and kinetics of Vβ4^+^ CD8^+^ T cell expansion. 8–12 week old IFNγR-/- C57Bl/6 mice were intranasally infected with 1x10^5^ pfu MHV68 (WT or M1st) or were mock infected and sacrificed at indicated times (n = 1–13 mice/group from one or two independent experiments). Right and accessory lobes were harvested and assessed for hydroxyproline content at days 4, 9, 28, and 90 post-infection. Statistics were performed using Mann-Whitney 2 tailed test to compare WT and M1st (* *P* = 0.0159, *** *P* = 0.0008).

To better understand the timing and development of pulmonary fibrosis in IFNγR-/- C57Bl/6 mice, we analyzed the extracellular matrix (ECM) content in lung tissue following infection, using the hydoxyproline assay. Lung tissue was collected at days 4, 9, 28, and 90 post-infection and assayed for collagen content. Consistent with our previous observations, we show that fibrotic scaring, as indicated by ECM deposition, fails to occur in the absence of M1 expression. Interestingly, we noted that timing of collagen deposition in the lung roughly mirrors that of the Vβ4^+^ CD8^+^ T cell expansion—suggesting an association between these phenomenon (Fig 7) [33].

### IFNγR-deficient mice which fail to develop an M1-dependent Vβ4^+^ CD8^+^ T cell expansion are protected from MHV68 induced fibrosis

**Fig 8:**
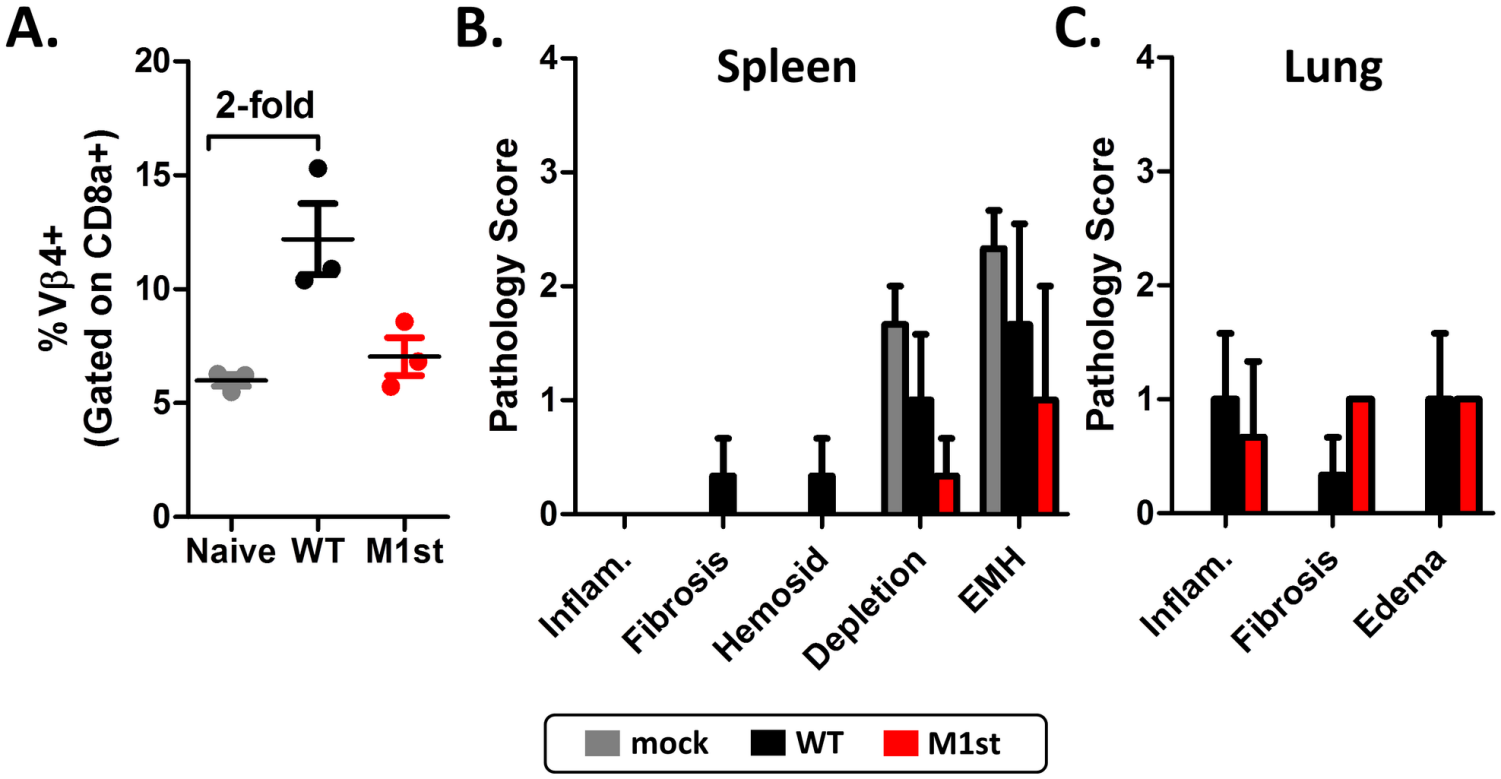
IFNγR-/- Balb/c mice are protected from MHV68 induced fibrosis. 8–12 week old IFNγR-/- Balbc mice were intranasally infected with 1000 pfu MHV68 (WT or M1st) or mock were infected and sacrificed at 28 days post infection (n = 3 mice/group). (A) Vβ4^+^ CD8^+^ T cell populations in spleen were assessed by flow cytometry. (B and C) Pathology scores were determined from H&E stained sections and fibrosis was evaluated with Masson’s trichrome stained tissue sections.

The level of M1-mediated Vβ4^+^ CD8^+^ T cell expansion that occurs during MHV68 infection varies in different mouse strains. C57Bl/6 mice have high levels of expansion, with an ca. 9-12-fold increase above naïve Vβ4^+^ CD8^+^ T cell levels; whereas Balb/c mice have very minimal expansion resulting in an ca. 2-4-fold increase in Vβ4^+^ CD8^+^ levels [33]. To evaluate the impact of Vβ4^+^ CD8^+^ T cell activation and expansion in MHV68 infection-induced fibrosis, we generated IFNγR-/- mice on the Balb/c background and infected these mice with either WT or M1st virus (Fig 8).

An unexpected finding, following the generation of the IFNγR-/- Balb/c mice, was the loss of Vβ4^+^ T cells in approximately 50% of naïve mice (S3 Fig). This loss of Vβ4^+^ T cells occurred in both the CD4^+^ and CD8^+^ T cell populations (S3A and S3B Fig), and appeared to occur during thymic selection as exiting single positive cells showed loss of Vβ4 (data not shown). To determine whether this loss resulted in other changes in the T cell repertoire, T cell receptor analysis was carried out to assess the frequencies of other Vβ subsets in mice that lost or maintained their Vβ4^+^ T cells. We found that the loss of Vβ4 expression in CD4^+^ T cells resulted in a compensatory increase in Vβ8.1/8.2, Vβ8.3, Vβ10b, and Vβ13 positive cells (S4 Fig). Loss of Vβ4 expression in CD8^+^ T cells, led to a compensatory increase in Vβ8.1/8.2, and Vβ8.3, and Vβ10b positive cells (S4 Fig). Due to this finding, mice were screened prior to infection to ensure the presence of Vβ4^+^ T cells for subsequent experiments.

As expected we observed a weak Vβ4^+^ CD8^+^ T cell expansion, resulting in an ca. 2-fold increase compared to naïve levels in the IFNγR-/- Balb/c mice (Fig 8A). Importantly, the modest M1-dependent Vβ4^+^ CD8^+^ T cell expansion in Balb/c mice failed to elicit cytokine production in response to recombinant M1 protein (S5 Fig). To assess fibrotic effects induced by MHV68 infection of IFNγR-/- Balb/c mice, spleen and lung were collected and assessed for pathological changes. Importantly, we failed to observe splenic atrophy in MHV68 infected mice in this background. Histological analysis of Masson’s trichrome stained tissue sections revealed little evidence of fibrosis in this background for both WT and M1st infected mice (Fig 8B and 8C). As such, there remains a strong correlation between expansion and activation of Vβ4^+^ CD8^+^ T cells and virus induction of fibrosis.

### Depletion of CD8 T cells prevents fibrotic disease

The CD8 T cell response plays an important role in clearance of MHV68 infection from the lung of wild type C57Bl/6 mice [27]. Additionally, the role of CD8^+^ T cells in fibrotic disease has been highlighted in IFNγR-/- mice. Depletion of CD4^+^ or CD8^+^ T cells was shown to reduce splenic pathology in IFNγR-/- 129/Sv/Ev mice [10]. These studies showed that depletion of CD8^+^ T cells led to restoration of spleen appearance, increased cellularity, and an overall reduction in MHV68 infectious centers [10]. However, it is worth noting that a striking difference exists between the 129/Sv/Ev and C57Bl/6 backgrounds; IFNγR-/- mice in the 129/Sv/Ev background undergo an apparent resolution of fibrosis at ca. 45 days post-infection [11] whereas C57Bl/6 mice fail to resolve disease. Nonetheless, the studies by Dutia and colleagues highlight an important role for T cells in development of fibrotic disease in IFNγR-/- mice [10].

**Fig 9:**
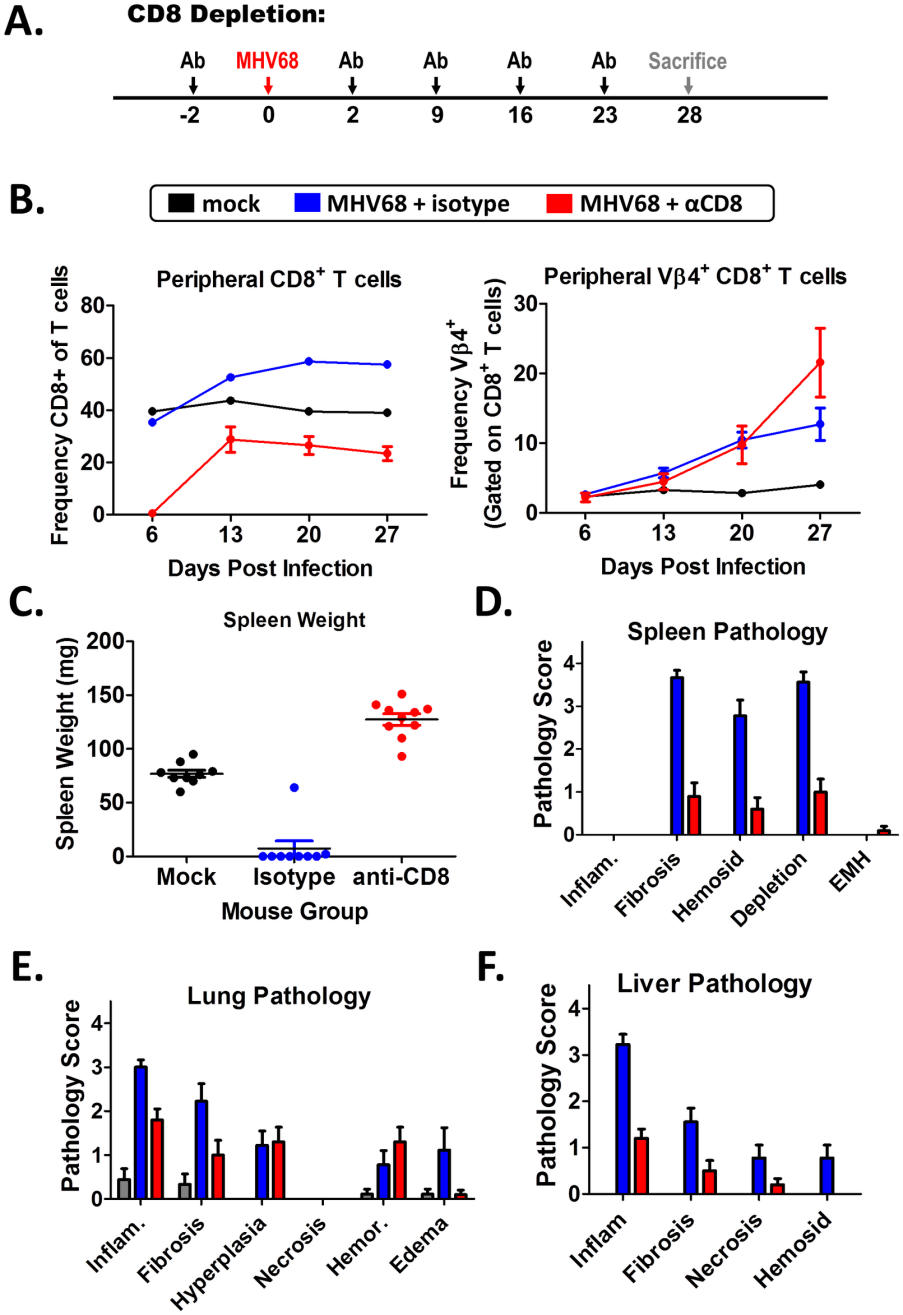
Depletion of CD8 T cells ameliorates MHV68 induced fibrotic disease in IFNγR-/- C57Bl/6 mice. 8–12 week old IFNγR-/- C57Bl/6 mice were intranasally infected with 1x10^5^ pfu WT MHV68 and treated with either Rat IgG isotype or Rat αCD8 (clone YTS 169.4) antibodies prior to sacrifice (n = 4–5 mice/group). (A) CD8 depletion strategy is shown. (B) Peripheral blood was assessed for CD8^+^ and Vβ4 ^+^ CD8 ^+^ T cell frequencies. (C) Mouse spleen weight at sacrifice and (D-F) pathology scores are shown. Scores were determined from H&E stained sections and fibrosis was evaluated with Masson’s Trichrome stained tissue sections.

As the M1st virus mutant lacks expansion of Vβ4^+^ CD8^+^ T cells, and exhibits reduced numbers of CD8^+^ T cells and viral antigen specific CD8^+^ T cells in the lungs of infected IFNγR-/- C57Bl/6 mice (Fig 6), we wanted to evaluate what role CD8^+^ T cells (including Vβ4^+^ CD8^+^ T cells) play in development of fibrosis. A technical limitation for Vβ4^+^ CD8^+^ T cell depletion had been noted in previous attempts to deplete this population [20, 35]. The single commercially available Vβ4 antibody, clone KT4, failed to deplete Vβ4^+^ T cells *in vivo* and instead simply masked detection by flow cytometry [20]. Due to this shortcoming, we were unable to specifically deplete Vβ4^+^ CD8^+^ T cells, and instead utilized a global CD8^+^ T cell depletion strategy. Mice were injected starting at day -2 before infection using a rat monoclonal anti-CD8 antibody (or an isotype control) (Fig 9A).

To measure the efficiency of depletion, weekly blood samples were collected and assessed by flow cytometry (Fig 9B). We observed a striking elimination in peripheral CD8^+^ T cells within the first week following treatment, and though these cells partially recovered—they remained ca. 50% below isotype treated levels through the course of infection (Fig 9B). Surprisingly, activated CD8^+^ T cells (CD62L^lo^ CD44^hi^) appeared to resist depletion while naïve populations (CD62L^hi^ CD44^lo^) were susceptible (data not shown). Due to this limitation, and the effector memory phenotype of Vβ4^+^ CD8^+^ T cells [20], we were unable to efficiently deplete the Vβ4^+^ CD8^+^ T cells that accumulate during infection (Fig 9B)—although it should be noted that since overall CD8 levels at later times post-infection only recovered to < 50% of isotype control antibody treated mice, that there was an ca. 50% reduction in Vβ4^+^ CD8^+^ T cells.

While there was only a partial depletion of peripheral CD8+ T cells using this strategy, we observed striking phenotypic changes in the infected mice. Compared to the isotype treated group, mouse body condition was greatly improved. Additionally, gross anatomic changes were apparent. For example, the atrophied spleen (Fig 9C) and irregular liver surface containing multifocal confluent raised nodules (data not shown), was prevented in the CD8^+^ T cell depleted group. Histological evaluation revealed reduced fibrosis, hemosiderosis, and cellular depletion in the spleen (Fig 9D); reduced inflammation, fibrosis, and edema in lung (Fig 9E); and reduced inflammation, fibrosis, necrosis, and hemosiderosis in the liver (Fig 9F) following CD8^+^ T cell depletion. Overall, these data point to a critical role of CD8^+^ T cells in mediating the pathology observed in the lung, spleen and liver.

## Discussion

An association between human herpesvirus (HHV) infection and IPF development and/or disease exacerbation is supported by a significant body of work. Some of the most compelling evidence for this association was reported in 2003 by Tang *et al*., where 97% of lung tissues from IPF patients were shown to have one or more HHV compared to 36% of control samples. Further, two or more herpesviruses (CMV, EBV, and HHV-8) were identified in 57% of IPF patients compared to 8% of controls [36]. Associations for EBV infection in IPF are supported by the observation of elevated levels of EBV DNA in the lung [37], presence of the EBV lytic spliced gene product WZhet in lung biopsies [38], and increased viral capsid antibody titers [39] (reviewed in [40]). Further, EBV infection of type II alveolar epithelial cells and has been shown to induce TGFβ production [24], while EBV protein LMP-1 has been shown to play a role in epithelial to mesenchymal transition [41]. Additionally, a recent report from Kropski et al. [41] suggested a role for ongoing herpesvirus replication in development of fibrotic disease. Asymptomatic relatives of patients with familial interstitial pneumonia were shown to have elevated endoplasmic reticulum stress in alveolar epithelial cells, lung remodeling, and epithelial dysfunction in association with herpesvirus replication [42]. However, not all studies have shown a correlation between EBV and IPF [43, 44]. Risk factors for IPF are numerous involving genetic, environmental, and infectious agents, and these risk factors need not all be present to result in disease [40, 45]. HHV are perhaps just one of the factors setting the stage for fibrotic disease.

Many similarities between the MHV68 model system in IFNγ unresponsive mice and human IPF have been described, including infection and apoptosis of alveolar epithelial cells, type II pneumocyte hyperplasia, epithelial to mesenchymal transition, and M2 macrophage differentiation in the lung [12–14]. Furthermore, IPF patients have been shown to shed equivalent levels of EBV from saliva and lower airways, while non IPF controls show a 10-fold reduction in titers from the lower airway [46]. This chronic infection in the lung is mirrored by the high levels of persistent MHV68 in IFNγR-/- C57Bl/6 lung as late as 90 days post infection, shown here and in previous studies [15, 17]. It is likely that viral persistence has many deleterious effects on the host. During this chronic stage of infection, repeated insult to the lung epithelium likely contributes to the ongoing damage and repair cycles. It was therefore noteworthy that we observed elevated levels of viral persistence with the fibrosis impaired M1-null mutant MHV68 (Fig 4C). We suggest that a cumulative influence, where viral persistence alone is not sufficient to induce fibrotic response in the absence of a strong inflammatory response.

A frequently disputed aspect of pulmonary fibrosis is the role of inflammation. Often, inflammation is not observed in lung biopsies from IPF patients (Reviewed [47]). Human trials utilizing anti-inflammatory treatments, such as corticosteroids, have shown no survival benefit, and chronic use is associated with increased co-morbidities [48, 49]. Further, the use of azathioprine, an immunosuppressant which blocks T and B cell proliferation as well as reducing levels of circulating monocytes and granulocytes, has failed to result in a survival benefit [49]. The strongest evidence contradicting the role of inflammation as a driver of fibrotic disease was revealed in the recent PANTHER-IPF study, in which anti-inflammatory treatment was actually harmful to IPF patients. In this study the triple treatment arm using N-acetyl cysteine, prednisone, and azathioprine had to be discontinued due to a 10% increase in mortality—largely due to respiratory causes, and ca. 3-fold increase in hospitalization and adverse effects [50]. This disparity between inflammation and disease is a confounding feature mirrored in many small animal models for IPF. Certainly bleomycin treatment, one of the most commonly used models for lung fibrosis, is initiated by a strong inflammatory response. These data have led to the hypothesis that an initiating inflammatory insult leads to epithelial damage and initiation of the tissue repair process, while 'multiple hits' may be required to induce fibrotic disease (discussed in [4, 45]).

Our understanding of the role T cells play in IPF is also complicated. T cells are a well-documented finding in the lungs of IPF patients, and are frequently found in areas of interstitial fibrosis in the lung [51–54]. Some researchers have shown an association between CD8 T cells and worsening clinical symptoms or disease progression [55–57]; while other studies have indicated CD4 T cells [58] and their activation [59] negatively impact disease status. Contradicting the aforementioned data, a recent report from Herazo-Maya and colleagues indicated that markers of T cell activation and signaling corresponded to a better prognosis [60]. However, there is a paucity of data showing a direct role of T cells in IPF. One obstacle in these studies is the fact that evaluation of human samples can only provide correlative insights at a snapshot during the course of disease (discussed [61]). Luzina *et al*. described T cells as an important component cell type of inflammation—likely affecting fibrosis through a diverse set of mechanisms—though not the driving force of IPF, perhaps these T cells modulate the severity of fibrosis [61]. Further adding to this complexity, small animal studies have also shown contradicting results for roles for T cells in pulmonary fibrosis. SCID or nude mice, both lacking T cells, have been shown to develop fibrosis in the bleomycin model [62, 63]. While models of pulmonary fibrosis utilizing asbestos have suggest a protective role of T cells in pulmonary fibrosis, where SCID and nude mice develop higher levels of fibrosis compared to WT mice, and immune reconstitution with T cells reduces fibrotic response [64]. Further, studies in CD28 knock out mice showed attenuated fibrosis, and adoptive transfer of CD28^+^ T cells is capable of restoring normal fibrotic response [65]. Additionally, in a model utilizing CCL18 overexpression, prolonged perivascular and peribronchial T cell infiltrate associated with collagen deposition is observed in the lung [66]. Collectively, these data suggest that T cells may wear many hats, functioning as profibrotic or antifibrotic mediators depending on the host environment.

Here we describe an immunological characterization of MHV68 infection of IFNγR-/- C57Bl/6 mice which results in fibrotic disease. This chronic infection leads to multi-organ fibrosis and has been utilized to evaluate the role of gammaherpesviruses in the development of pulmonary fibrosis. Through examination of a fibrosis-deficient virus, MHV68 M1st, our study has been able to highlight the role of CD8^+^ T cells in the development of pulmonary fibrosis in this model. We have shown that this viral mutant, which fails to elicit expansion and activation of Vβ4^+^ CD8^+^ T cells, has reduced levels of viral antigen specific CD8^+^ T cells in the lungs. We suggest that the absence of the Vβ4^+^ CD8^+^ T cell population results in reduced inflammation and cellular infiltrate in the lung, protecting mice from subsequent immunopathology and fibrosis. We show that mice lacking a robust Vβ4^+^ CD8^+^ T cell expansion are protected from fibrotic disease, highlighting a critical role for this population of cells in disease progression. Furthermore, we show that depletion of CD8^+^ T cells during acute infection with the WT virus prevents the development of fibrosis, directly implicating CD8^+^ T cells in the fibroproliferative process. Future studies will be necessary to clarify the role of CD8^+^ T cells, and the impact of the Vβ4^+^ CD8^+^ T cell expansion. It will be of interest to evaluate how CD8 depletion impacts viral replication and cellular infiltrates in the lung. Additionally, understanding which CD8 subsets are required for disease—including epitope specific, bystander activated, and Vβ4 –may shed light on the disease process.

We find it notable that a striking immunologic change revealed by these analyses was the increased levels of neutrophils in the lungs of WT MHV68 infected mice (Fig 3C). Several studies have implicated neutrophil involvement in human IPF [67–69] and pulmonary fibrosis in murine models [70–73]. We therefore postulate that the effector CD8^+^ T cells found during MHV68 infection, which express high levels of TNFα and potentially GM-CSF, may effect neutrophil activation and survival (as described in [74]). It will be of interest to evaluate the chemokine production from CD8^+^ T cells in the absence and presence of M1 expression during MHV68 infection.

Finally, with respect to the role of herpesviruses in IFP, an open label study showed that ganciclovir treatment resulted in improvement in 9/14 IPF patients [75]. During this study, treatment was given for two weeks and patients were assessed for changes in EBV antibody titers, steroid use, and forced lung vital capacity. Though encouraging, there were several limitations to this study—including absence of a control arm, and small sample size, necessitating further exploration. Animal models have also shown successful treatment of fibrosis with antiviral cidofovir. Mora and colleagues showed that cidofovir treatment was able to reduce fibrotic disease in the MHV68 infected IFNγR-/- C57Bl/6 with treatment given as late as 60 days post infection- well after the onset of fibrotic disease [15]. Certainly gaining a better understanding of how gammaherpesviruses influence the immune response, and what features are critical for disease, may lead to more streamlined treatments.

The conflicting data on the role of viral infections, as well as the role of CD8^+^ T cells, may point toward distinct mechanisms for development of lung fibrosis. Here we have provided additional data which is consistent with a role for MHV68 M1-driven expansion and activation of Vβ4^+^ CD8^+^ T cells as mediators in inflammation and fibrotic disease in IFNγR-/- mice. These data support to the role of CD8^+^ T cells as an important contributor in fibrotic pathology. Furthermore, we highlight a potential target for therapeutic intervention; in cases were herpesvirus infection is playing a role in disease progression and may be managed pharmacologically or immunologically.

## Materials and Methods

### Ethics statement

This study was carried out in strict accordance with the recommendations in the Guide for the Care and Use of Laboratory Animals of the National Institutes of Health. The protocol was approved by the Emory University Institutional Animal Care and Use Committee and in accordance with established guidelines and policies at Emory University School of Medicine (Protocol Number: YER-2002245-031416GN).

### Mice

Six to eight week old female C57Bl/6 and Balb/c mice were obtained through Jackson Laboratory (Bar Harbor, ME) and IFNγR-/- C57Bl/6 and Balb/c mice were bred in-house. Mice were infected at eight to twelve weeks of age. Prior to infection mice were sedated with isofluorane and intranasally infected with 5x10^5^pfu in 20ul of DMEM. Mice used in T cell depletion studies were sedated with isofluorane and injected intraperitoneally with 100μg Rat anti-CD8 (clone YTS 169.4), a generous gift of Arash Grakoui and Elizabeth Elrod, or Rat IgG2b (clone LTF-2) as an isotype control according to experimental schedule described in Fig 9.

### Pathology

Tissues were collected and fixed in 10% buffered neutral formalin for up to 7 days prior to processing for hematoxylin and eosin or Masson’s trichrome staining. Samples were sent for evaluation by pathologists, blinded samples were scored by AG and imaging was performed by CLC. Pathology scores are as follows 0 = Normal, 1 = 1–2 foci, 2 = 3–4 foci, 3 = Multifocal, 4 = Diffuse.

### Tissue preparation

Following euthanasia, tissues were collected in cold complete medium. Spleens were processed mechanically, and lung tissue was minced and digested using type IV Collagenase and DNAse I to produce a single cell suspension. Subsequently, single cell suspensions were treated with red blood cell lysis buffer and cells were enumerated.

### Flow cytometry

Single cell suspensions were resuspended in PBS supplemented with 2% fetal bovine serum and 2mM EDTA. Samples were blocked with CD16/32 and stained using standard procedures, antibodies used for these studies are described in Table 1. Events were recorded on BD LSRII flow cytometer and results were analyzed using FloJo software (Tree Star Inc). Tetramer staining was performed using MHV68 peptide specific tetramers, p79/H-2K^b^ and p56/H-2D^b^, generated by the NIH Tetramer Core at Yerkes National Primate Research Center at Emory University. For intracellular cytokine staining (ICCS) eBioscience fixable viability dye (catalog # 65-0865-14) was used.

**Table 1:** Antibodies used for flow cytometry.

Staining	Marker	1° Ab Fluor	Vendor	Clone
**Innate Lung Cells**	CD11b	BV421	BD Pharmingen	M1/70
	MHCII	V500	BD Horizon	M5/114.15.2
	CD45	FITC	BD Pharmingen	30-F11
	CD103	PerCP-Cy5.5	Biolegend	2E7
	CD64	PE	Biolegend	X54-5/7.1
	Siglec F	PE-CF594	BD Horizon	E50-2440
	CD11c	PE Cy7	BD Pharmingen	HL3
	CD24	APC	BD Pharmingen	M1/69
	Ly6G	Alexa700	BD Pharmingen	1A8
	LyGC	APC-Cy7	BD Pharmingen	AL-21
**Tetramer Staining**	CD62L	Pacific Blue	Biolegend	MEL-14
	CD8a	Pacific Orange	Invitrogen	3B5
	CD45	PerCP-Cy5.5	BD Pharmingen	30-F11
	CD44	PE-Cy7	BD Pharmingen	IM7
	CD3e	Alexa700	BD Pharmingen	500A2
**ICCS Lung Cells**	CD8	Pacific Orange	Invitrogen	3B5
	CD4	PerCP-Cy5.5	BD Pharmingen	RM4-5
	CD3e	PE-Cy7	eBioscience	145-2C11
	IL2	BV421	Biolegend	JES6-5H4
	IFNg	APC	eBioscience	XMG1.2
	TNFα	PE	Biolegend	MP6-XT22
	VB4	FITC	BD Pharmingen	KT4
**Vβ4 Analysis IFNgR-/- Balb/c**	CD8	Pacific Orange	Biolegend	MEL-14
	CD4	Pacific Blue	BD Pharmingen	RM4-5
	VB4	FITC	BD Pharmingen	KT4
	VB5	PerCP-eFluor710	eBioscience	MR9-4
	CD62L	APC	Biolegend	MEL-14
	CD44	PE	Biolegend	X54-5/7.1
	CD19	APC-Cy7	BD Pharmingen	ID3
	CD3e	PE-Cy7	eBioscience	145-2C11
**Vβ4 Analysis CD8 Depletions**	CD8	Pacific Orange	Invitrogen	3B5
	CD4	PE	BD Pharmingen	RM4-5
	VB4	FITC	BD Pharmingen	KT4
	CD44	PE-Cy7	BD Pharmingen	IM7
	CD62L	APC	Biolegend	MEL-14
	CD3e	Alexa 700	BD Pharmingen	500A2
	CD19	APC-Cy7	BD Pharmingen	rmC5-3
**Fibrocyte Staining**	CD45	PerCP Cy5.5	BD Pharmingen	30-F11
	Col1	none	Rockland(Cat# 600-401-103S) ^†^, ^§^	N/A
**Macrophage Activation**	CD11b	BV421	BD Pharmingen	M1/70
	MHCII	V500	BD Horizon	M5/114.15.2
	CD45	FITC	BD Pharmingen	30-F11
	CD64	PE	Biolegend	X54-5/7.1
	CD24	PE CF594	BD Horizon	M1/69
	CD11c	PE Cy7	BD Pharmingen	HL3
	CD40	Alexa647	Biolegend	HM40-3
	CD206	Alexa647	AbD Serotec	MR5D3
	CD80	APC	Biolegend	16-10A1
	CD86	APC	BD Pharmingen	GL1
	CD71	APC	eBioscience	R17217
	RELMa	none	Peprotec(Cat # 500-P214) ^†^, *	N/A
**TCR (Vβ) Repertoire Analysis**	VB	FITC	BD Pharmingen(Cat # 51-0944L)	^‡^
	CD19	APC-Cy7	BD Pharmingen	ID3
	CD4	Pacific Blue	BD Pharmingen	RM4-5
	CD3e	PE	BD Pharmingen	17A2
	CD44	PECy7	BD Pharmingen	IM7
	CD8a	Pacific Orange	Invitrogen	3B5
**ICCS Splenocytes**	CD8	BV711	Biolegend	53–6.7
	CD3e	PE-Cy5	Biolegend	145-2C11
	CD44	PE-Cy7	BD Pharmingen	IM7
	IL2	BV421	Biolegend	JES6-5H4
	IFNg	APC	eBioscience	XMG1.2
	TNFα	PE	BD Pharmingen	MP6-XT22
	VB4	FITC	BD Pharmingen	KT4

Abbreviations used: ICCS- intracellular cytokine staining, D- Donkey, R-Rat, Rb-Rabbit.

^†^ Catalog number provided for items without specific clone information.

^‡^ Multiple antibody clones used, refer to manufacturer information.

^§^ DαR-PE from Jackson Immuno Research (Cat# 711-116-152) was used as a secondary antibody.

* DαRb-Alexa647 from Life Technologies (Cat# A31573) was used as a secondary antibody.

### Viral titers

Plaque assays were performed as previously described [76]. NIH 3T12 cells were plated in six-well plates 1 day prior to infection at 2×10^5^ cells per well. Organs were subjected to 4 rounds of mechanical disruption of 1 min each by using 1.0mm zirconia-silica beads (Biospec Products, Bartsville, OK) in a Mini-Beadbeater-8 instrument (Biospec Products). Serial 10-fold dilutions of organ homogenate were plated onto NIH 3T12 monolayers in a 200-μl volume. Infections were performed for 1h at 37°C with rocking every 15 min. Immediately after infection plates were overlaid with 1.5% methylcellulose in complete DMEM. After 10 days, cells were stained with 0.12% (final concentration) Neutral Red. The next day, methylcellulose was aspirated, and plaques were enumerated. The sensitivity of the assay is 50 PFU/organ.

### Viral persistence

A highly sensitive limiting dilution assay to determine viral persistence previously described [77] was used. Briefly, the left lung was homogenized in 1ml of complete DMEM media using 1.0mm Zirconia/Silicon beads (BioSpec products) 4 times (1 min homogenization, followed by 1 minute resting on ice). The single cell homogenate was lysed with 0.5mm Zirconica/Silicon beads using the same homogenization program. The final homogenate was then plated on Mouse Embryonic Fibroblasts (MEFs) in 2 fold dilutions, up to a total of 8 dilutions. Wells were incubated in a low evaporation incubator (37°C, 5% CO2) for a total of 14 days. Each well was then evaluated for cytopathic effect (CPE). Data is presented as percent of wells displaying CPE at each plated dilution.

### Assessment of active TGFβ

Mink lung epithelial cell line (MLEC clone 23) stably transfected with plasminogen activator inhibitor-1 fused to a luciferase reporter gene, a generous gift of Dr. Dan Rifkin, were plated at 5x10^5^ cells/well for infection in a 12 well plate. Cells were infected with MHV68-YFP or MHV68-M1st-YFP at different multiplicities of infection. At 42 hours post infection, cell lysates were collected and assessed for luciferase activity using a TD-20/20 luminometer.

### Western blot

Lung tissues from infected C57Bl/6 mice were collected and homogenized using 200ul of Sigma CelLytic MT Mammalian Tissue Lysis/Extraction reagent per 40mg lung tissue. Following lysis membranes were pelleted at 14,000xg for 10 minutes and samples were stored at -80°C. 30μg of cell lysate was loaded on a denaturing 12% SDS polyacrylamide gel and detected with TGFβ1 antibody (Ebioscience clone A75-2), membranes were stripped and probed for β-actin.

### Hydroxyproline assay

Mice were euthanized and right and accessory lobes of the lung were collected and frozen at -80°C until further processing as previously described [78]. Briefly, lung tissue was homogenized in 1mL of PBS, and hydrolyzed by the addition of 1ml of 12N hydrochloric acid (HCl). Samples were then baked at 110°C for 12 h. Aliquots (5μl) were then assayed by adding chloramine T solution for 20 min followed by development with Erlich’s reagent at 65°C for 15 min. Absorbance was measured at 550 nm, and the amount of hydroxyproline was determined against a standard curve generated using known concentrations of hydroxyproline standard (Sigma).

### Simulation for T cell effector function

WT or IFNγR-/- mice on the C57Bl/6 were infected with 1x10^5^pfu WT or M1st MHV68 intranasally. Mice were sacrificed at 28 days post infection and single cell suspensions of lung cells were prepared as described above. Cells were either unstimulated or stimulated with 20ng/ml PMA & 1μg/ml ionomycin, 10μM viral peptides p56 (AGPHNDMEI) or p79 (TSINFVKI) a generous gift of Dr. Mandy Ford, or recombinant M1 or M1st protein containing supernatants previously described in [20]. Cells were cultured in the presence of 1μl/ml BD-GolgiPlug (brefeldin-A) and respective stimuli for 4–6 hours at 37°C. Cells were subsequently surface stained, fixed and permeablized using BD Cytofix/Cytoperm, and stained for intracellular cytokines. For antibodies used, refer to Table 1.

### Analysis of Vβ repertoire

Naïve WT or IFNγR-/- mice were sacrificed and single cell suspensions of splenocytes were prepared using standard methodology. Cells were then blocked with CD16/32 and stained using BD-Pharmingen Mouse Vβ TCR Screening Panel in FITC along antibodies listed in Table 1.

## Supporting Information

S1 Fig Elevated levels of innate cell populations are observed in IFNγR-/- C57Bl/6 mice in the presence of M1 expression. 8–12 week old IFNγR-/- C57Bl/6 mice were intranasally infected with 1x10^5^ pfu MHV68 (WT or M1st) and sacrificed at indicated times post infection (n = 3–4 mice/group at each time-point). Whole lungs were harvested and assessed for cellular composition by flow cytometry using a panel to detect innate immune cell populations (described in [21]) and fibrocytes (described in [25]). Statistics were assessed using Mann-Whitney 2 tailed test, ** *P* = 0.0048.
(TIF)

S2 Fig Elevated levels of alternative macrophage activation are observed in lung of fibrotic IFNγR-/- C57Bl/6 mice. 8–12 week old C57Bl/6 IFNγR-/- mice were intranasally infected with 1x10^5^ pfu MHV68 (WT or M1st) and sacrificed at indicated times post infection (n = 3–4 mice/group at each timepoint). Whole lungs were harvested and assessed for macrophage population and phenotype. (A-B) Absolute number of alveolar and interstitial macrophages were quantified and assessed for alternative activation using RELMα and CD206 expression.
(TIF)

S3 Fig IFNγR deficiency leads to deletion of Vβ4^+^ T cells from about half of the IFNγR-/- Balb/c mice. 7–10 week old naïve WT or IFNγR-/- Balb/ mice were assessed for Vβ4^+^ T cell populations in peripheral blood by flow cytometry. (A-B) Quantitation of CD4 or CD8 T cell populations are shown alongside representative figures of WT or IFNγR-/- Balb/c that have either retained or lost the Vβ4^+^ T cells. IFNγR-/- Balb/c (n = 35) WT Balb/c (n = 5).
(TIF)

S4 Fig Loss of Vβ4 population does not substantially alter T cell repertoire in IFNγR-/- Balb/c mice. T cell repertoire was evaluated in 15 week old naïve IFNγR-/- Balb/c mice. Spleens were harvested and frequency of Vβ subsets were assessed. (A) CD4 and (B) CD8 T cells is shown. IFNγR-/- Balb/c (n = 8) WT Balb/c (n = 5).
(TIF)

S5 Fig Lack of M1-induced cytokine response from Vβ4^+^CD8^+^ T cells in Balb/c mice. WT C57Bl/6 (filled symbols) and Balb/c mice (opened symbols) were intranasally infected with 1000 pfu MHV68 (WT or M1st) or left naïve and sacrificed at 28 dpi (n = 2–3 mice/group). Splenocytes were isolated for *in vitro* stimulation. Frequency and absolute number of cells are shown for M1 recombinant protein stimulated cells. Total CD8 T cells (A) and Vβ4^+^ CD8^+^ T cells (B) are shown. Samples gated on Vβ4^+^ CD8^+^ T cells show IFNγ (C) and TNFα (D) producing cells following stimulation with recombinant M1 protein.
(TIF)
