# Supplementary figures and images for: CD8+ T Cell Response to Gammaherpesvirus Infection Mediates Inflammation and Fibrosis in Interferon Gamma Receptor-Deficient Mice
Source: PLoS One. 2015 Aug 28;10(8):e0135719. doi: 10.1371/journal.pone.0135719 (PMC4552722; doi:10.1371/journal.pone.0135719)

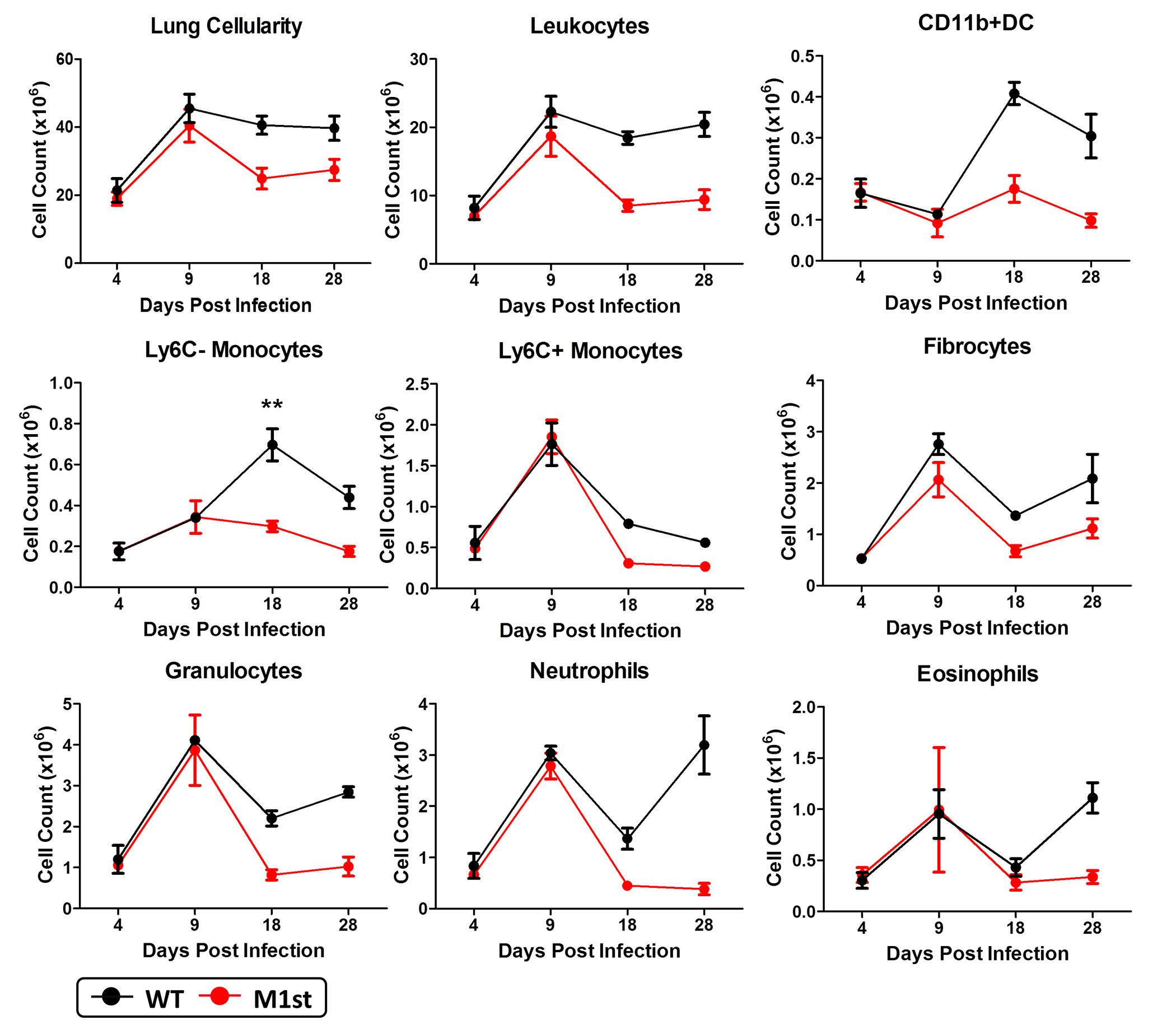

Supplement: S1 Fig — 8–12 week old IFNγR-/- C57Bl/6 mice were intranasally infected with 1x10^5^ pfu MHV68 (WT or M1st) and sacrificed at indicated times post infection (n = 3–4 mice/group at each time-point). Whole lungs were harvested and assessed for cellular composition by flow cytometry using a panel to detect innate immune cell populations (described in [21]) and fibrocytes (described in [25]). Statistics were assessed using Mann-Whitney 2 tailed test, ** P = 0.0048. (TIF) [file pone.0135719.s001.TIF]

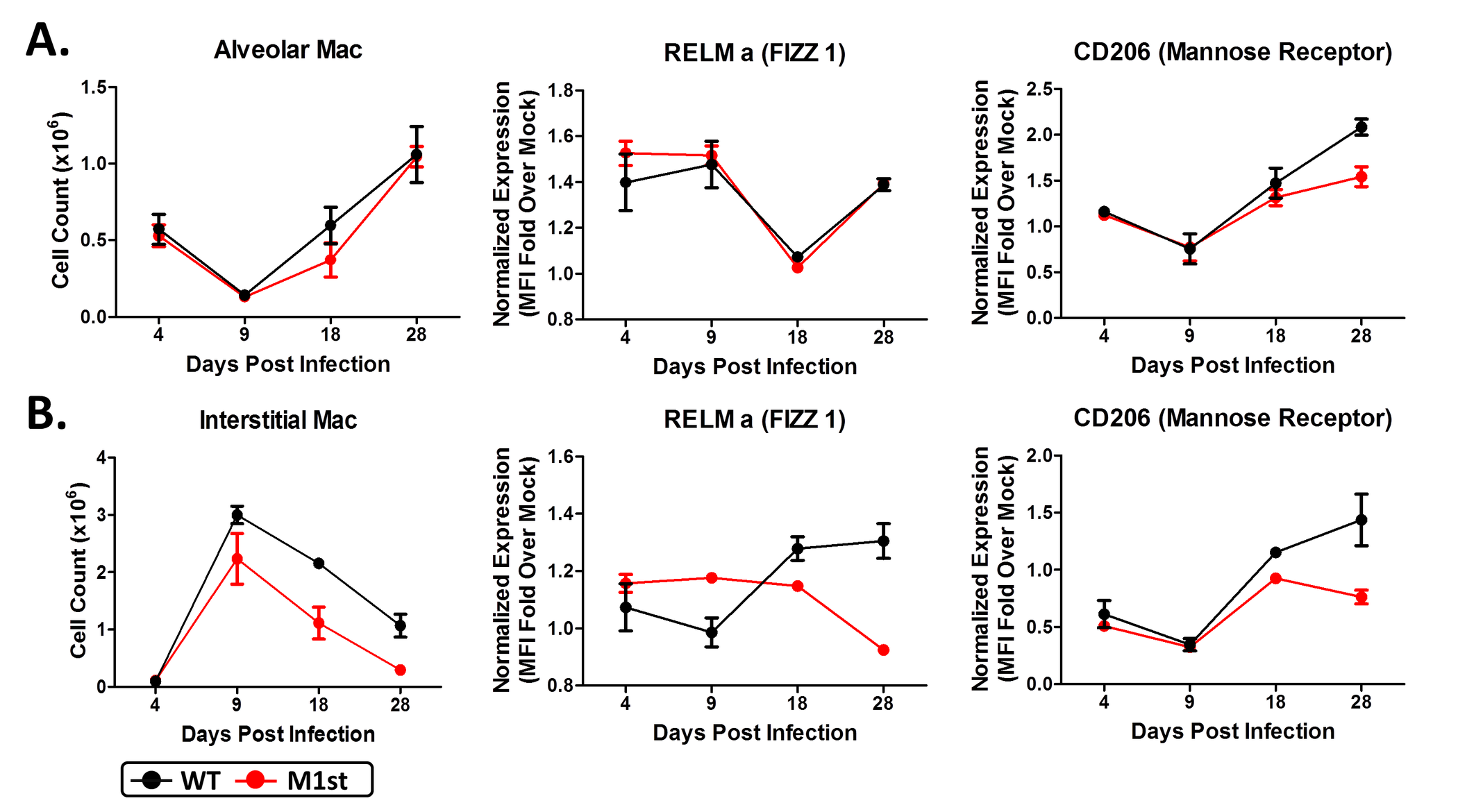

Supplement: S2 Fig — 8–12 week old C57Bl/6 IFNγR-/- mice were intranasally infected with 1x10^5^ pfu MHV68 (WT or M1st) and sacrificed at indicated times post infection (n = 3–4 mice/group at each timepoint). Whole lungs were harvested and assessed for macrophage population and phenotype. (A-B) Absolute number of alveolar and interstitial macrophages were quantified and assessed for alternative activation using RELMα and CD206 expression. (TIF) [file pone.0135719.s002.TIF]

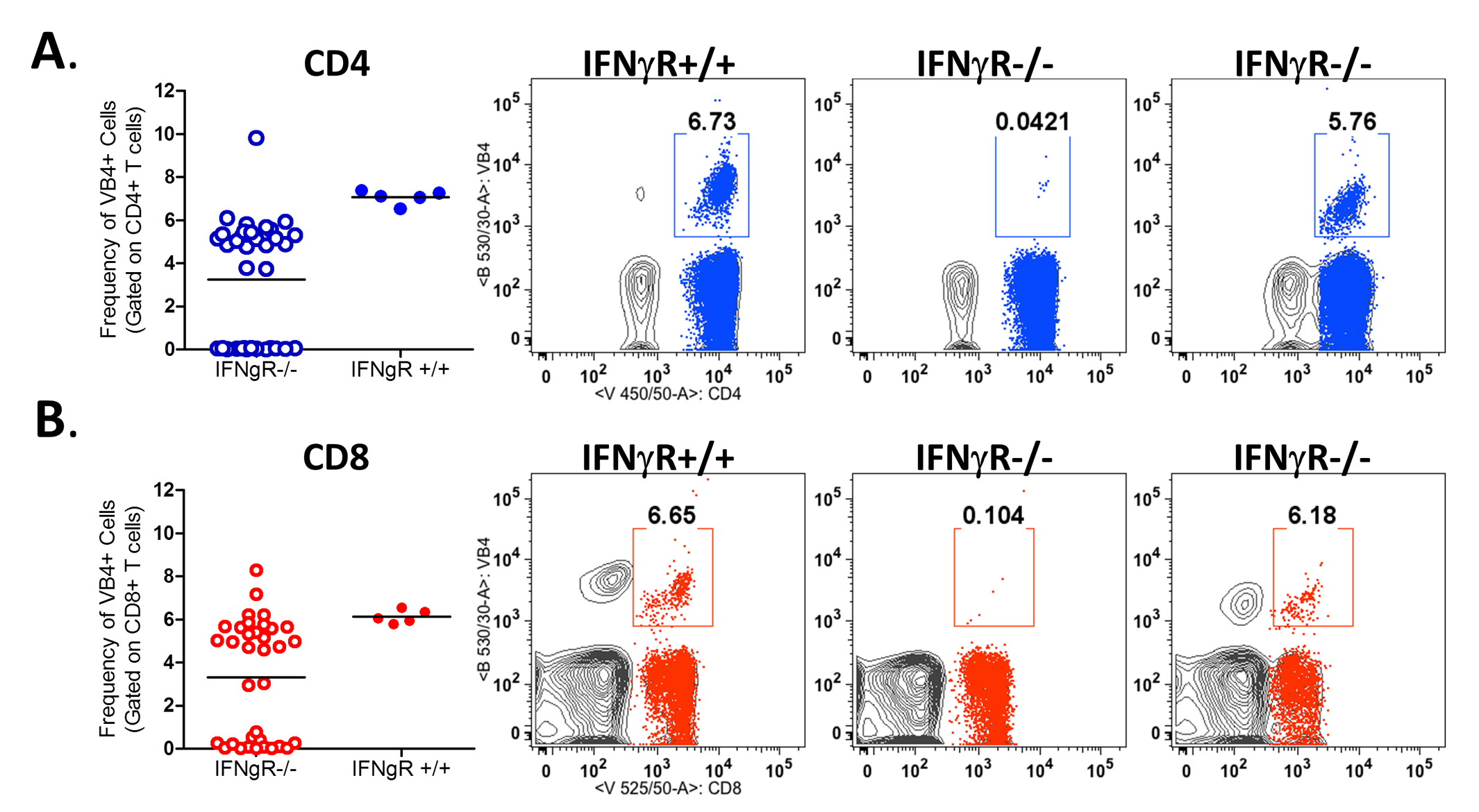

Supplement: S3 Fig — 7–10 week old naïve WT or IFNγR-/- Balb/ mice were assessed for Vβ4+ T cell populations in peripheral blood by flow cytometry. (A-B) Quantitation of CD4 or CD8 T cell populations are shown alongside representative figures of WT or IFNγR-/- Balb/c that have either retained or lost the Vβ4+ T cells. IFNγR-/- Balb/c (n = 35) WT Balb/c (n = 5). (TIF) [file pone.0135719.s003.TIF]

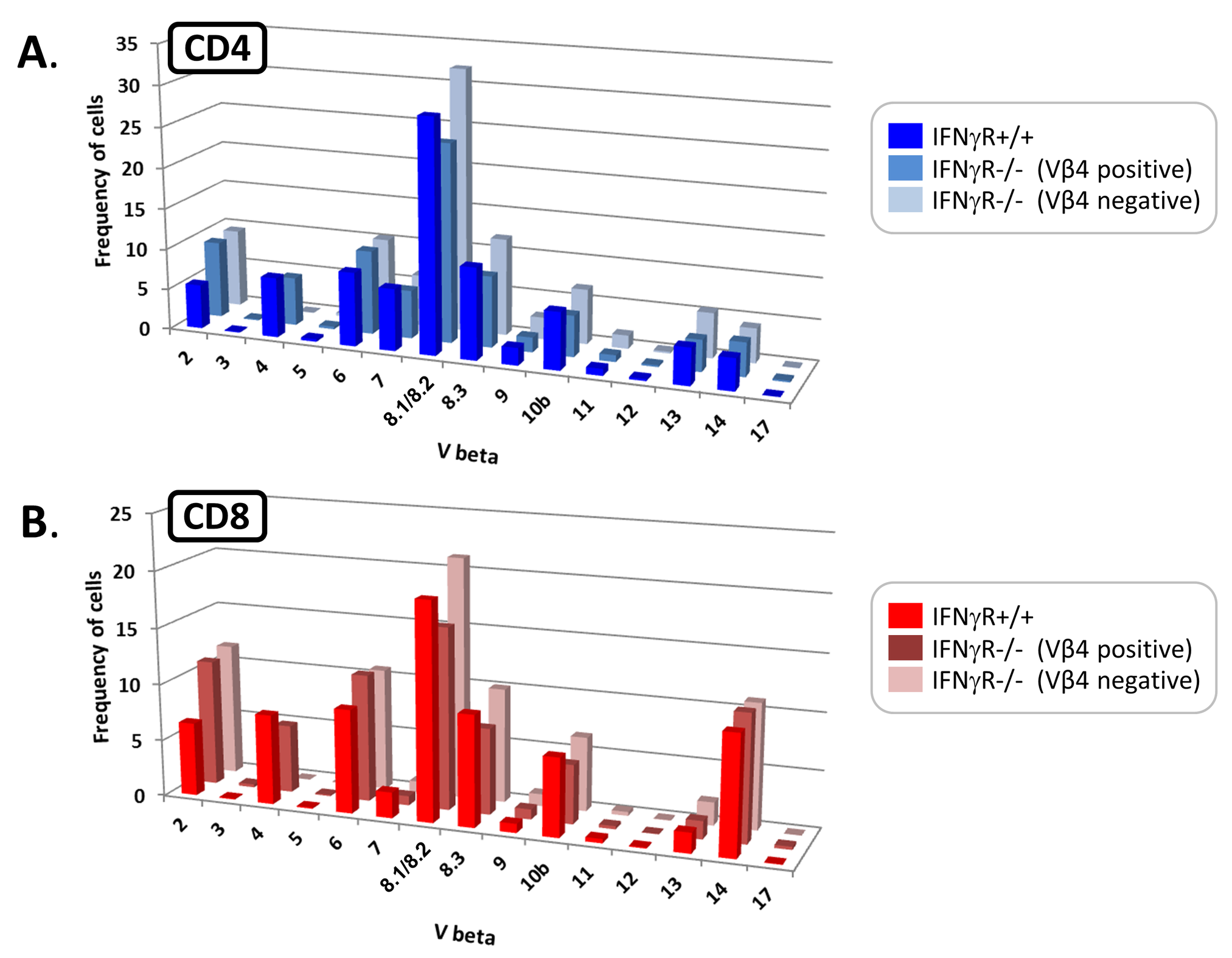

Supplement: S4 Fig — T cell repertoire was evaluated in 15 week old naïve IFNγR-/- Balb/c mice. Spleens were harvested and frequency of Vβ subsets were assessed. (A) CD4 and (B) CD8 T cells is shown. IFNγR-/- Balb/c (n = 8) WT Balb/c (n = 5). (TIF) [file pone.0135719.s004.TIF]

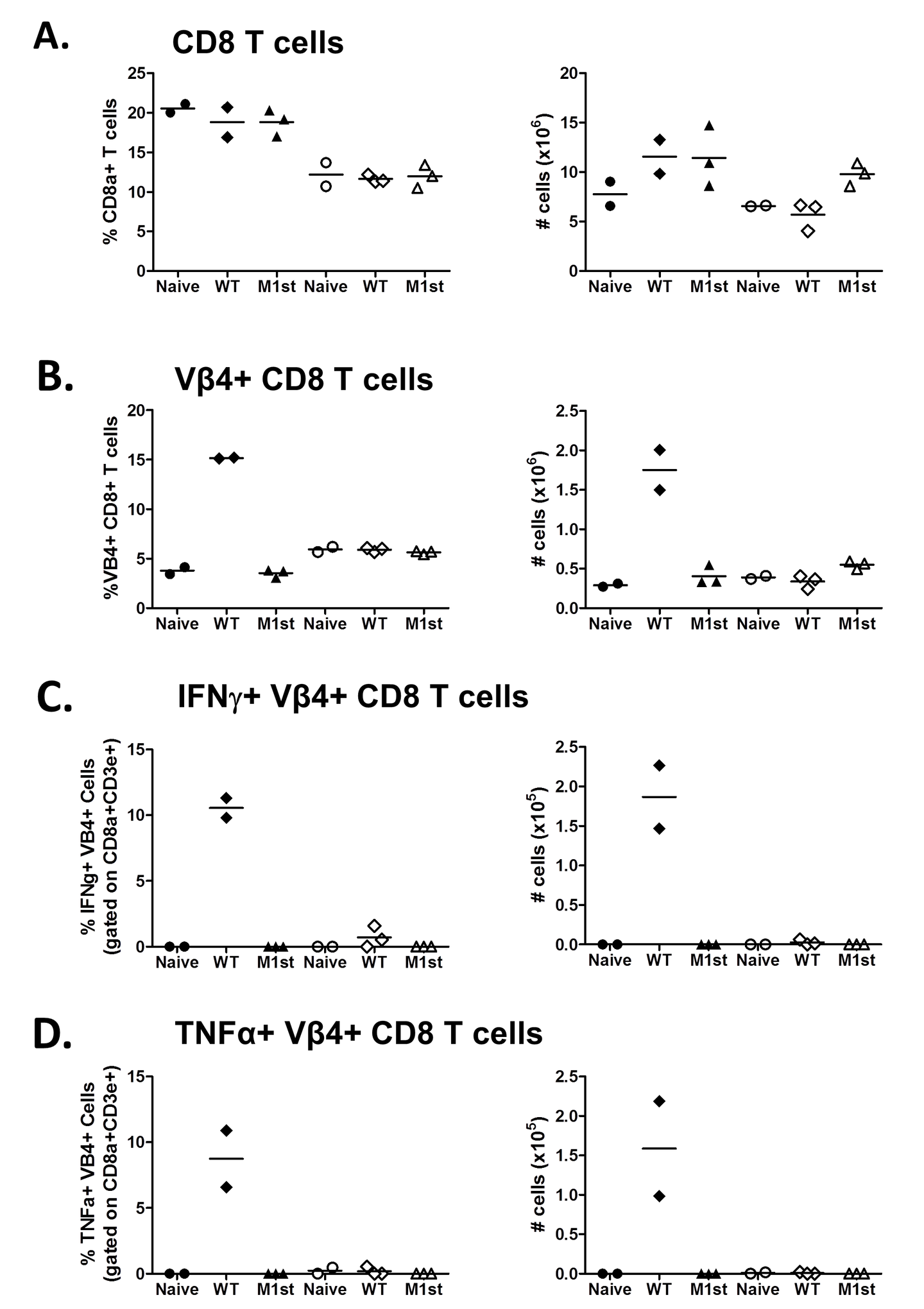

Supplement: S5 Fig — WT C57Bl/6 (filled symbols) and Balb/c mice (opened symbols) were intranasally infected with 1000 pfu MHV68 (WT or M1st) or left naïve and sacrificed at 28 dpi (n = 2–3 mice/group). Splenocytes were isolated for in vitro stimulation. Frequency and absolute number of cells are shown for M1 recombinant protein stimulated cells. Total CD8 T cells (A) and Vβ4+ CD8+ T cells (B) are shown. Samples gated on Vβ4+ CD8+ T cells show IFNγ (C) and TNFα (D) producing cells following stimulation with recombinant M1 protein. (TIF) [file pone.0135719.s005.TIF]
